# Sex-specific impact of early life stress on adult lung inflammatory response after LPS and Poly I:C exposures

**DOI:** 10.1016/j.bbih.2026.101204

**Published:** 2026-02-23

**Authors:** Karine Bouchard, Dany Patoine, Joanny Roy, Stéphanie Fournier, David Marsolais, Richard Kinkead, Jean-François Lauzon-Joset

**Affiliations:** a Centre de Recherche de l’IUCPQ - Université Laval, Québec, QC, Canada; b Departement de Médecine, Faculté de Médecine, Université Laval, Québec, QC, Canada; c Departement de Pédiatrie, Faculté de Médecine, Université Laval, Québec, QC, Canada

**Keywords:** Early life stress, Maternal separation, Inflammatory response, Innate immunity, Macrophages, Biological sex differences

## Abstract

Biological sex influences the development and function of the immune system, shaping responses to infection through both innate and adaptive mechanisms. Early life stress can disrupt immune development through long-term changes in the hypothalamic-pituitary-adrenal (HPA) axis. Neonatal maternal separation (NMS) is an established model of early life adversity that mimics theses effects with sex-specific effects on physiological outcomes. To study how NMS alters immune responses to infection, newborn rats were separated from their mother for 3 h per day from post-natal day 3 to 12, whereas controls were undisturbed. Lung immune response was evaluated at 8 weeks old using LPS, which models gram-negative bacteria infection, and Poly I: C mimicking viral infection; both inducing activation of innate immune cells that play a role in the activation of adaptive immune response. Immune cell populations of broncho-alveolar lavage, lungs and spleen were measured by flow cytometry. In addition to sex differences of inflammatory responses, NMS increased broncho-alveolar lavage neutrophilia after Poly I:C and LPS exposure in males. Furthermore, accumulation of macrophages, neutrophils and natural killer cells in the airways of NMS animals was modulated in a sex- and stimulus-specific fashion. In addition, NMS also induced systemic immune modulation, as observed by the increased proportion of spleen NK cells in NMS male. Thus, our data suggest that early life stress exerts sexually dimorphic effects on immune cells and increases the risk of respiratory tract infections later in life.

## 1 Introduction

### 1.1 Stress response

Stress can arise from controllable or uncontrollable situations, which induce a natural coping response that can modify the state of homeostasis (Oyola et al., 2017; Charmandari et al., 2005; Juruena et al., 2020). Stress responses are adaptation mechanisms, including a multitude of complex physiological reactions, all of which can have a major impact on immunity and inflammation (Juruena et al., 2020; Young et al., 2013; Robb et al., 2016). The impact of stress on immunity depends on the nature, intensity and time of exposure to stressors. In adults, acute stress is associated with an increased inflammatory response due to innate/non-specific immune activation, which is more pronounced in women compared to men (Segerstrom et al., 2004). On the other hand, persistent stressors are associated with an increased risk of infections and immunosuppression (Lyons et al., 2023).

In children, stressful situations have a long-term influence on brain development and the hypothalamic-pituitary-adrenal axis (HPA), including the corticotropic axis, and disrupt the immune response (Cole et al., 2012). In addition, fetal *in utero* stress, which is derived from maternal stress, is a well-established risk factor for preterm birth and is associated with multiple adverse neonatal outcomes, including low birth weights, increased susceptibility to certain infections and lasting immunological changes (Beijers et al., 2014). When stressful events occur during childhood, such as child abuse, maltreatment, parental separation and illness, they can have major repercussions that persist until adulthood (Juruena et al., 2020; Juruena, 2023), especially for the immune system, which is developing in this critical phase. Although there is growing evidence that childhood stress/adversity and sexual dimorphism are linked to increased susceptibility to immune-related diseases later in life (Oyola et al., 2017; Cole et al., 2012), the underlying mechanisms are still poorly understood.

### 1.2 Immune response

The immune system is a complex network comprising innate and adaptive immune cells. Innate immune cells are the first line of defense in response to pathogens and alarmins. Their responses are non-specific and orchestrated by different cell subsets, including macrophages, neutrophils and natural killer cells (NK) (Robb et al., 2016; Nicholson, 2016; Wechsler et al., 2021). Accumulation of macrophages and neutrophils at the site of infection is a hallmark of inflammatory amplification (Aegerter et al., 2022). During respiratory infection, alveolar macrophages (AM) are the first cells to respond by detecting and phagocytosing viral or bacterial particles as well as apoptotic cells. AM have pattern recognition receptors that recognize pathogen particles, leading to the secretion of pro-inflammatory cytokines and chemokines that initiate the immune response (Turvey et al., 2010; Herold et al., 2015). On the other hand, adaptive immune cells, including T and B cells, are cornerstones of antigen-specific response and take longer to accumulate in the infected site. T and B cells are mainly activated in secondary lymphoid organs, namely the lymph nodes and the spleen (H et al., 2019). During viral infection, CD4^+^ T cells (helper) produce inflammatory cytokines and promote the production of specific antibodies by B cells, while CD8^+^ T cells (cytotoxic) kill infected cells (Kwiecień et al., 2021; Chen et al., 2018). Regulatory T cells play a role in limiting the activation of adaptive immunity by producing anti-inflammatory cytokines, such as IL-10 (Nicholson, 2016).

In addition, biological sex is a major factor in the development of the immune system, as it contributes to physiological differences in the response following exposure, recognition, clearance and transmission of microorganisms, including bacteria and viruses (Carroll et al., 2010; Klein et al., 2016). In females, innate immune cells, such as macrophages and dendritic cells, respond to infection with a more robust production of anti-viral mediators, such as interferon, compared to males (Gal-Oz et al., 2019). In addition, neutrophil activation and antibody production are higher in females compared to males (Klein et al., 2016). Opposingly, during infection, male innate immune cells produce more IL-10 (Torcia et al., 2012).

To understand the impact of early life stress on immune function development, animal models of neonatal maternal separation (NMS) are often used, as they reproduce several sex-specific pathophysiological adaptations observed in humans (Vetulani, 2013; Genest et al., 2004; Gulemetova et al., 2011). Loss of maternal contact by the newborns causes them distress, without altering their growth (Liu et al., 1997). Early life stress causes physiological alterations in pups, such as heart rate and hormone levels (Vetulani, 2013). Furthermore, adult rats exposed to maternal separation in their youth present persistent hyperactivity of the HPA axis and exhibit anxious behavior (Vetulani, 2013; Kalinichev et al., 2002; Hofer, 1975). LPS mimics gram-negative bacteria, whereas Poly I: C mimics viral infection, and both induce activation of innate immune cells that play a role in the activation of adaptive immune response. Thus, the present study was designed to explore the sex-specific relationship between infection and early life stress, using rats exposed to NMS stimulated with either LPS or Poly I:C.

## 2 Materials and methods

### 2.1 Animals

Sprague Dawley rats (Charles River, Saint-Constant, QC, Canada) were kept in a specific pathogen-free environment (CRIUCPQ-Université Laval, Québec, QC, Canada). Experiments, housing and care procedures were approved by the Committee of Animal Care of Université Laval according to guidelines of the Canadian Council on Animal Care.

### 2.2 Stress model and experimental treatments

All animals used were born in our animal care facilities (CRIUCPQ-Université Laval, Québec, QC, Canada). Studs and dams were purchased from Charles River and bred in-house. Experiments were performed on 66 control and 90 NMS females and males rats. Animals were assigned randomly to experimental groups. Following birth, pups were separated from their mother for 3 h per day (7h30-10h30 a.m.) from post-natal day 3 to 12 (Fig. 1A). Separated pups were placed in a temperature (35 °C) and humidity (45%) controlled incubator and isolated from each other by a board partition (Genest et al., 2004; Gulemetova et al., 2011). On day 21, rats were weaned and housed (2 per cage) under standard animal care conditions until they reached sexual maturity (8-9 weeks old). Those animals were compared with undisturbed animals (CTRL), which were not subjected to the NMS procedure and maintained under standard animal care procedures (Genest et al., 2004; Lehmann et al., 2000). Adult rats were instilled intranasally under light isoflurane anesthesia with 10 mg of LPS or 100 mg of Poly I:C diluted in 200 ml phosphate-buffered saline (PBS). Since it is known that PBS doesn't lead to inflammation, baseline groups didn't receive PBS (Boston et al., 2003). Animals for multiple litters were randomly assigned to each groups. Twenty-four hours following LPS or Poly I:C exposure, rats were euthanized by ketamine/xylazine overdose. Bronchoalveolar lavage (BAL) and tissues were harvested.

**Fig. 1:**
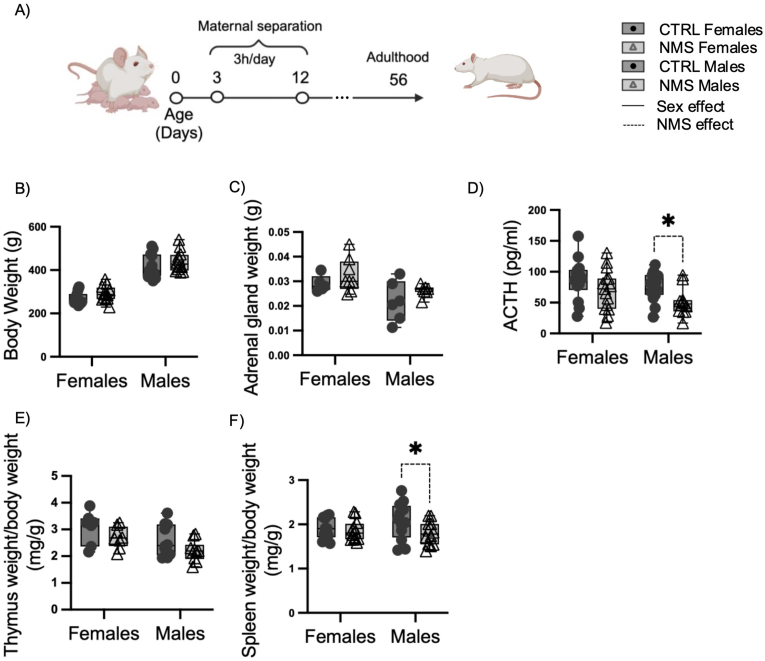
NMS decreased the size of lymphoid organs and levels of ACTH hormones in plasma of males, but did not alter body weight or adrenal gland size. Experimental design of neonatal maternal separation (NMS) (A). At young adulthood (56 days), no difference in body weight (B) and adrenal gland weight (C) were measured between CTRL and NMS groups. The level of adrenocorticotropic hormone (ATCH) in plasma were lower in NMS males (D). The thymus (E) and spleen (F) weights were lower in NMS males. Non-parametric two-way analyses of variance (ANOVA) followed by multiple comparison test (Fisher's LSD test) were used. ∗Significantly different from corresponding group (∗p < 0.05). For each graph n = between 8 and 18 rats.

### 2.3 Assessment of plasma adrenocorticotropic hormone levels

Blood was collected with a syringe inserted into the abdominal aorta and transferred into EDTA coated tubes. Plasma was separated by centrifugation and frozen at −80 °C until assayed. The adrenocorticotropic hormone (ATCH) level was quantified by multiplex kit (Millipore, Merck KGaA, Darmstadt, Germany) according to manufacturer's instructions.

### 2.4 Tissue preparation and flow cytometry analyses

Single-cell suspension of lungs and spleens was obtained using digestive enzymes (Collagenase 1 mg/ml and DNase 0.2 mg/ml) (Reichard et al., 2019). Lung and spleen single-cell suspensions, and BAL cells (Bouchard et al., 2023) were stained for flow cytometry analysis using the following antibodies: CD3 (1F4), RT1B (OX-6), CD172a (OX-41), CD11b (WT-5), CD43 (W3/13), CD4 (W3/25), CD161a (10/78), CD45R (HIS24), CD8a (OX-8), CD45 (OX-1) (BD Biosciences, San Jose, CA; Biolegend, San Diego, CA). Data were acquired using Diva-driven LSRFortessa (BD Biosciences) and analyzed with FlowJo 10.10.0 software (BD Biosciences).

### 2.5 Statistics

Statistical analyses were performed using GraphPad Prism software (Version 10.4.0 (527) for MacOS). Two-way analyses of variance (ANOVA) (Values of the source of variation are presented in Sup Table 1) followed by multiple comparison test were used as indicated in figure legends. The significance threshold was set to P < 0.05. Data are presented as box plot (min. to max.).

## 3 Results

### 3.1 NMS does not impact adrenal gland weights in adults, but influences male spleen, thymus size and ACTH level in plasma

Although males had a higher body weight than females (Fig. 1B), there was no significant difference in body and adrenal gland weights between NMS and undisturbed (CTRL) rats (Fig. 1B–C). The activation of the HPA axis was evaluated by measuring ACTH plasma levels. ACTH plasma level was lower in NMS males compared to CTRL males, but unchanged in NMS females (Fig. 1D). NMS male thymus tend to weigh less compared to NMS female rats (Fig. 1E). Moreover, NMS male spleens weigh less than those of CTRL male (Fig. 1F). There was no significant difference in thymus and spleen weight between female CTRL and NMS. Given that adults who experienced NMS had sex specific alterations of lymphoid organ sizes and ACTH plasma levels, we investigated the response of male and female NMS rats to inflammatory stimuli.

### 3.2 Increased inflammatory response in bronchoalveolar lavage in male NMS after LPS exposure

**Fig. 2:**
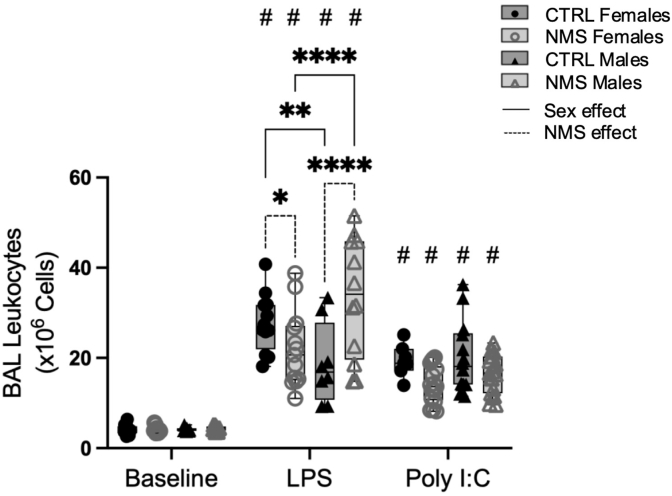
BAL inflammatory response to LPS is more severe in NMS males. Adult males (closed symbols) and females (open symbols) who were exposed to early life stress (NMS) or not (CTRL) were challenged with LPS or Poly I:C intranasal exposure. Broncho-alveolar lavages were obtained 24 h after, and total cell counts were measured. Non-parametric two-way analyses of variance (ANOVA) followed by multiple comparison test (Fisher's LSD test) were used. ∗Significantly different from corresponding group (∗p < 0.05; ∗∗p < 0.005). #Significantly different from baseline groups (p < 0.05). For each graph n = between 7 and 21 rats.

**Fig. 3:**
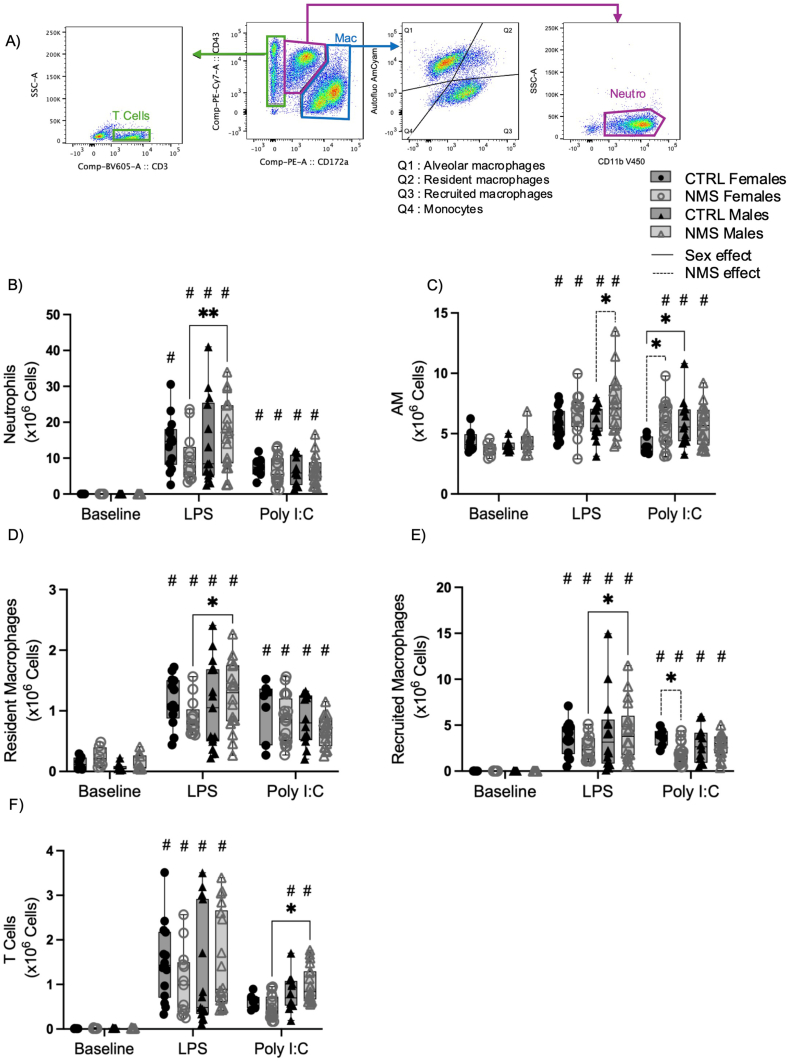
BAL macrophage inflammatory response is altered in NMS males and females. Broncho-alveolar lavages of NMS and CTRL rats were obtained 24 h after LPS or Poly I:C exposure, and cellular composition was assessed by flow cytometry (A). Neutrophils (B), AM (C), resident macrophages (D), recruited macrophages (E) and T cells (F) were identified. Non-parametric two-way analyses of variance (ANOVA) followed by multiple comparison test (Fisher's LSD test) were used. ∗Significantly different from corresponding group (∗p < 0.05; ∗∗p < 0.005; ∗∗∗∗p < 0.0001). #Significantly different from baseline groups (p < 0.05). For each graph n = between 7 and 21 rats.

Lung inflammation was assessed in response to LPS or Poly I:C exposure and severity was measured in bronchoalveolar lavage (BAL) samples 24 h post-exposure in young adults. At baseline, there were no significant difference in the number of immune cells found in BAL between the experimental groups (Fig. 2). After LPS and Poly I:C exposure, all groups displayed a strong increase in total BAL cells compared to baseline (Fig. 2). Biological sex also played a role in recruitment of immune cells since it was higher in CTRL females than CTRL males after LPS exposure (Fig. 2). Interestingly, NMS increased immune cell recruitment in response to LPS in males but not in females (Fig. 2). Although Poly I:C exposure caused the accumulation of leukocytes in BAL compared to baseline (Fig. 2), there was no significant difference in total number of immune cells between experimental groups (Fig. 2). Subsequently, we investigated immune cell subsets present in BAL using flow cytometry (Fig. 3A).

LPS-induced inflammatory response in BAL was mostly dominated by neutrophils (Fig. 3B), whereas Poly I:C response was characterized by a mix of neutrophils and recruited macrophages (Fig. 3). After LPS exposure, NMS males had a higher number of neutrophils compared to NMS females (Fig. 3B). Although this difference was not observed after Poly I:C exposure (Fig. 3B), the proportion of neutrophils in the BAL after Poly I:C increased in NMS males and decreased in NMS females compared with their respective Poly I:C CTRL group (Sup. Fig. 1).

The number of alveolar macrophages (AM) was higher in NMS males compared to CTRL males after LPS exposure (Fig. 3C), whereas after Poly I:C exposure, the number of AM was significantly increased in CTRL males and NMS females compared to CTRL females (Fig. 3C). NMS increased the number of resident macrophages in males compared to females after LPS exposure (Fig. 3D). The number of recruited macrophages in NMS females was lower after exposure to Poly I:C than in CTRL females (Fig. 3E). In addition, NMS males had a higher number of T cells than NMS females after Poly I:C exposure only (Fig. 3F).

### 3.3 NMS modulates innate and adaptive immune cells in the lung

**Fig. 4:**
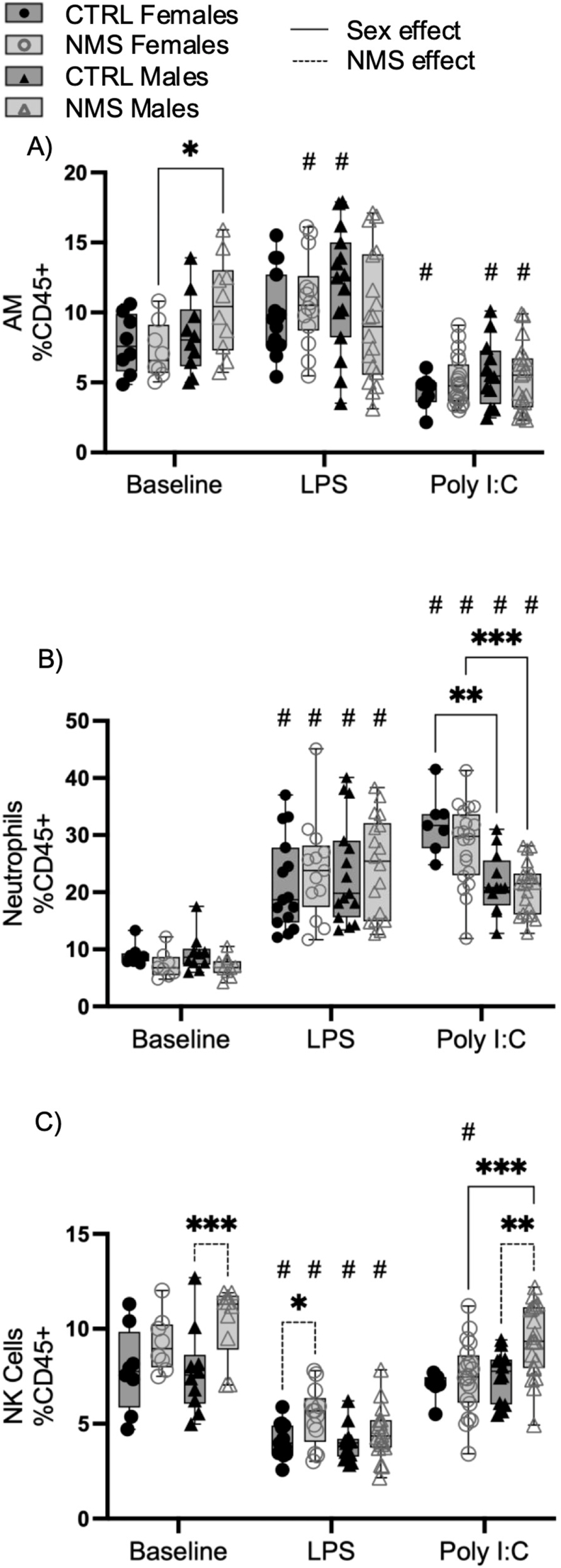
Lung natural killer cells showed sex-specific inflammatory recruitment after LPS and Poly I:C exposure. Lung cell suspensions from NMS and CTRL rats were prepared 24 h after LPS or Poly I:C exposure. AM (A), neutrophils (B) and NK (C) were identified by flow cytometry. Non-parametric two-way analyses of variance (ANOVA) followed by multiple comparison test (Fisher's LSD test) were used. ∗Significantly different from corresponding group (∗p < 0.05; ∗∗p < 0.005; ∗∗∗p < 0.0005; ∗∗∗∗p < 0.0001). #Significantly different from baseline groups (p < 0.05). For each graph n = between 7 and 21 rats.

Given the difference in BAL inflammatory response, we then investigated whether or not cell subsets were also impacted within the lung tissue itself (Fig. 4). At baseline, lung proportion of AM was higher in NMS males than in NMS females (Fig. 4A), whereas the proportion of lung NK cells was only elevated in NMS males (Fig. 4C). Lung inflammatory responses after both LPS and Poly I:C exposures wwere mostly characterized by an increased proportion of neutrophils compared to baseline (Fig. 4B). After Poly I:C exposure, CTRL and NMS females had a higher proportion of neutrophils than, respectively, CTRL and NMS males (Fig. 4B). After LPS exposure, NMS females had a higher proportion of NK cells, whereas after exposure to Poly I:C, NMS males have a higher proportion of NK cells than CTRL males and NMS females (Fig. 4C).

**Fig. 5:**
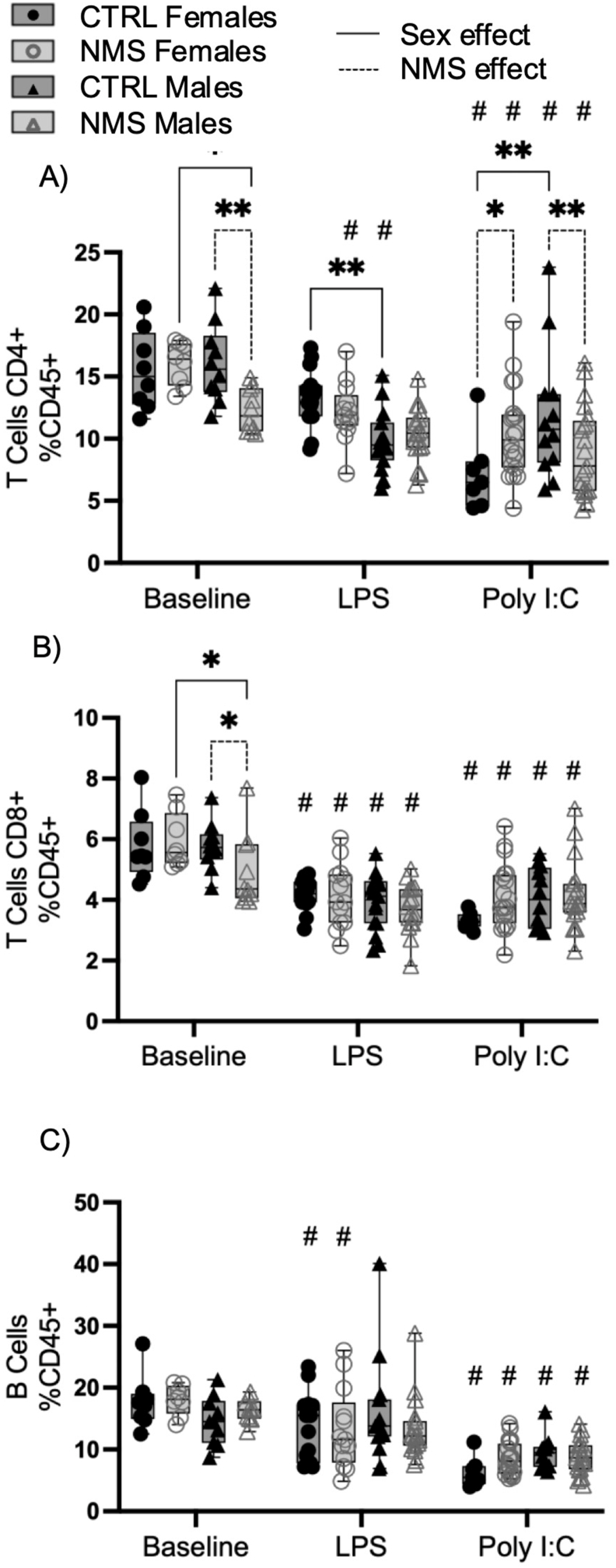
Lung CD4^+^ T cell proportion is modulated by NMS after Poly I:C exposure. Lung cell suspensions from NMS and CTRL rats were prepared 24 h after LPS or Poly I:C exposure. CD4^+^ T cells (A), CD8^+^ T cells (B) and B cells (C) were identified by flow cytometry. Non-parametric two-way analyses of variance (ANOVA) followed by multiple comparison test (Fisher's LSD test) were used. ∗Significantly different from corresponding group (∗p < 0.05; ∗∗p < 0.005; ∗∗∗p < 0.0005; ∗∗∗∗p < 0.0001). #Significantly different from baseline groups (p < 0.05). For each graph n = between 7 and 21 rats.

We then characterized the lung adaptive arm of the immune response, where we observed a modulation of T and B cell subsets after LPS and Poly I:C exposures (Fig. 5). There was an effect of biological sex and NMS on T cell proportion at baseline. Specifically, NMS males had a lower proportion of CD4^+^ and CD8^+^ T cells than CTRL males and NMS females (Fig. 5A–B). After exposure to LPS, CTRL males had a lower proportion of CD4^+^ T cells than CTRL females (Fig. 5A), whereas after Poly I:C, CTRL males had a higher proportion of CD4^+^ T cells than CTRL females (Fig. 5A). Furthermore, after exposure to Poly I:C, NMS males had a lower proportion of CD4^+^ T cells than CTRL males, whereas NMS females had a higher proportion than CTRL females (Fig. 5A).

### 3.4 NMS modulates innate immune cell populations in lymphoid organs after LPS exposure

**Fig. 6:**
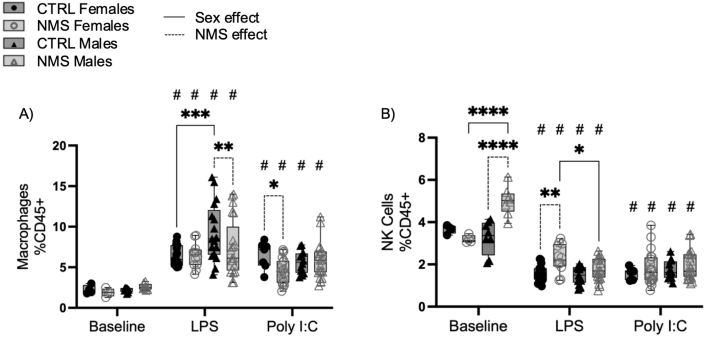
LPS and Poly I:C exposure modulated spleen innate immune response of male and female NMS animals. Splenocyte suspensions from NMS and CTRL rats were prepared 24 h after LPS or Poly I:C exposure. Macrophages (A) and NK cells (B) were identified by flow cytometry. Non-parametric two-way analyses of variance (ANOVA) followed by multiple comparison test (Fisher's LSD test) were used. ∗Significantly different from corresponding group (∗p < 0.05; ∗∗p < 0.005; ∗∗∗p < 0.0005; ∗∗∗∗p < 0.0001). #Significantly different from baseline groups (p < 0.05). For each graph n = between 7 and 21 rats.

To understand if the alteration of the lung immune responses by NMS was localized or systemic, we evaluated the immune cell composition of the spleen in response to lung exposure to LPS and Poly I:C, which was mostly comprised of an increased proportion of splenic macrophages (Fig. 6A). In the spleen, 24 h after LPS exposure, the proportion of macrophages was higher in CTRL males compared to CTRL females and NMS males (Fig. 6A). After Poly I:C exposure, there was a lower proportion of macrophages in NMS females compared to CTRL females (Fig. 6A). NMS males had a significantly higher proportion of NK cells at baseline compared to CTRL males and NMS females (Fig. 6B), whereas after LPS exposure, NMS females had a higher proportion of NK cells than CTRL females and NMS males (Fig. 6B).

**Fig. 7:**
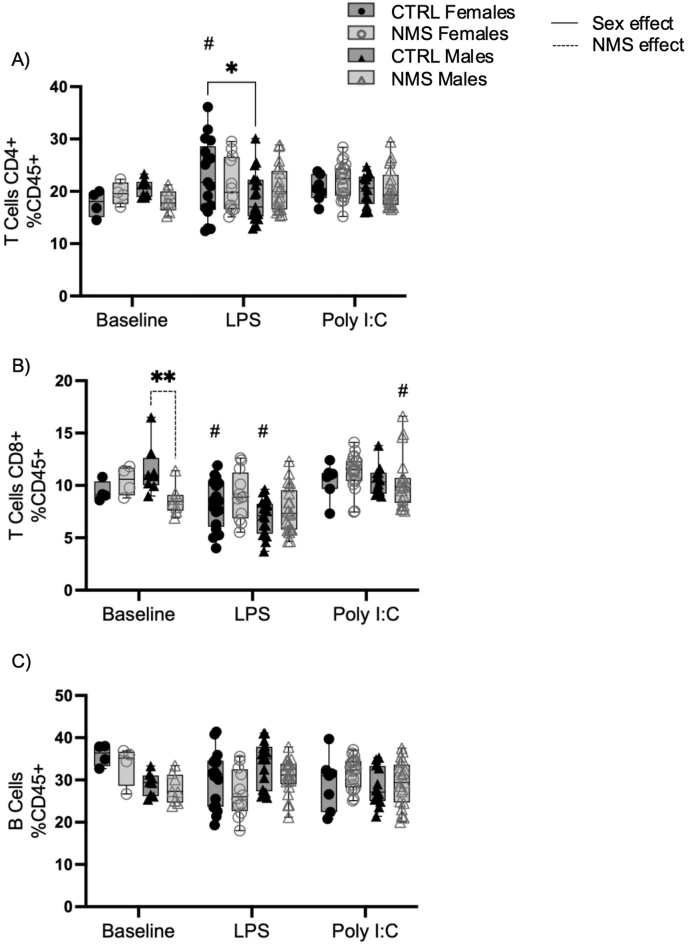
Spleen adaptive inflammatory response was mostly unchanged in NMS animals. Splenocyte suspensions from NMS and CTRL rats were prepared from spleen 24 h after LPS or Poly I:C exposure. CD4^+^ T cells (A), CD8^+^ T cells (B) and B cells (C) were identified by flow cytometry. Non-parametric two-way analyses of variance (ANOVA) followed by multiple comparison tests (Fisher's LSD test) were used. ∗Significantly different from corresponding group (∗p < 0.05; ∗∗p < 0.005; ∗∗∗p < 0.0005; ∗∗∗∗p < 0.0001). #Significantly different from baseline groups (p < 0.05). For each graph n = between 7 and 21 rats.

Next, we characterized spleen lymphocyte cell subsets, which were mostly unchanged either at baseline, and after LPS and Poly I:C exposure (Fig. 7), with the exception that at baseline, the proportion of CD8^+^ was higher in CTRL male compared to NMS males (Fig. 7B) and after exposure to LPS, the proportion of CD4^+^ T cells is higher in CTRL females compared to CTRL males (Fig. 7A).

## 4 Discussion

Epidemiological data demonstrate significant sex-based differences in the susceptibility and severity of bacterial and viral infections (Klein et al., 2016; Jaillon et al., 2019; Nunn et al., 2009). Men who contract a severe respiratory infection are more often hospitalized (Klein et al., 2016), which is linked with a higher viral load. In women, the elimination of viruses occurs more rapidly, but it is often associated with immunopathological damage (Carroll et al., 2010; Muenchhoff et al., 2014; Ghosh et al., 2017). Following infection, neutrophil activation in women is higher compared to men (Klein et al., 2016; Spitzer, 1999). Moreover, it is known that sex-specific difference in the innate immune system affects the early response to infections and can influence the following adaptive immune response (Dunn et al., 2024). Here, we observed a difference between NMS and CTRL males, but not in female groups. To the best of your knowledge, the origins of these sex-specific consequences of lung infections are still unclear. However, the data reported here indicate that exposure to stress during early life is an important determinant in those sex-based differences.

Early life stress can have profound sex-specific impacts on brain development, but its consequences on inflammatory response is less understood. Thus, using the neonatal maternal stress model allows to reproduce several sex-specific pathophysiological adaptations observed in humans (Vetulani, 2013), while inflammatory stimuli, LPS and Poly I:C mimic, respectively, gram-negative bacteria and viral infection. LPS and Poly I:C both induce activation of innate immune cells that play a role in the activation of the adaptive immune response. The severity of inflammatory responses were attested by the accumulation of immune cells in the BAL. Here, we demonstrated that NMS males have a higher number of immune cells in BAL, supporting that bacterial infection severity can be altered by stress mediators. For instance, stress can cause motility, growth and persistence of bacterial infection in animals (Kuriyama et al., 1996; Verbrugghe et al., 2012). Stress can also interfere with cytokine and chemokine production, maturation and differentiation of immune cells (Verbrugghe et al., 2012; Yang et al., 2000; Webster et al., 2002). Several aspects of the immune system can be influenced by stress hormones. In fact, several immune cells possess receptors for certain stress hormones, like ACTH and glucocorticoids, which could directly affect the immune response. Glucocorticoids can also modulate the transcription of proinflammatory and anti-inflammatory mediators (Verbrugghe et al., 2012; Webster et al., 2002).

In this study, we identified sex-specific differences in the impact of neonatal stress on immune response, more specifically, innate and early phase leukocyte mobilization. For instance, we showed that after LPS exposure, the number of neutrophils and AM were higher in NMS males. We also confirmed sexual dimorphism of lung inflammatory responses, exemplified by a higher proportion of neutrophils in females than males (CTRL and NMS). Moreover, we observed that early life stress disrupted innate lymphoid cells, especially the accumulation of NK cells in the lung, as well as a higher proportion of AM at baseline in NMS males. On the other hand, the proportion of NK cells was higher in NMS females after LPS exposure and in NMS males after Poly I:C exposure, suggesting that early life stress modulated immune responses in a sex-specific manner and according to the inflammatory stimuli.

While multiple inflammatory parameters were similarly altered by LPS and Poly I:C in NMS animals, inflammatory cell subsets accumulating in the airways of NMS animals were modulated in a sex- and stimulus-specific fashion, especially macrophages and natural killer cells. Therefore, sex differences can be linked to sexual hormone signalling, leading to recruitment of inflammatory cells. In males, testosterone is known to have immunosuppressive properties, leading to a reduction in recruitment of inflammatory cells (Card et al., 2006; Park et al., 2004). On the other hand, in females, estrogen appears to dampen LPS-driven inflammation, reducing neutrophil infiltration, whereas ovariectomy increases susceptibility to LPS-induced inflammation (Card et al., 2006). Female sex hormones can also enhance adaptive immunity and NK activation, whereas male androgens may dampen NK responsiveness (Bouman et al., 2005). After Poly I:C exposure in mice brain, males have a stronger interferon response than females, suggesting immune cell recruitment might be higher in males (Posillico et al., 2021). Therefore, antiviral response could play a role in NK cell activation that would differ between males and females (Posillico et al., 2021; Pujantell et al., 2023). Here, we have shown that NMS females have greater accumulation of NK cells, suggesting that this stress model modulated antiviral response in a sex-specific manner. Early-life stress via maternal separation may reprogram NK migration and cytotoxic patterns differently in males versus females, due to HPA-axis interference and altered antiviral signalling (Klein et al., 1995).

Previous anxiety-provoking experience can modify the immune system (Katz et al., 2025). During infections, cytokines can activate the HPA axis and increase glucocorticoid levels. Glucocorticoid hormones produced and secreted by the adrenal glands downregulate the HPA axis, reducing the expression of proinflammatory cytokines, suggesting that these hormones may prevent overactivation of the response to infection (Avitsur et al., 2006; Bailey et al., 2003; Blalock, 1994). Previous reports have demonstrated the sex-specific alteration of the HPA axis by early life stress (Genest et al., 2004; Liu et al., 1997). Glucocorticoid levels are influenced by prior adverse life experience and can influence activities of many immune cells, like NK cells and leukocyte trafficking (Eddy et al., 2014; Cain et al., 2017). The adrenal gland is an essential stress-responsive organ that is part of the HPA axis. Some studies have shown that rats exposed to chronic stress often exhibit adrenal enlargement (Gamallo et al., 1986; Martí et al., 1993; Ulrich-Lai et al., 2006). However, early life stress did not interfere with adrenal gland weight. Thymic atrophy is also linked with complications of environmental stressors like emotional stress that can activate the HPA axis to induce the production of the stress hormone cortisol, causing abrupt thymus involution (Haynes et al., 2000; Gruver et al., 2008; McEwen, 2003; Kioukia-Fougia et al., 2002). Here, we demonstrated that only male rats exposed to NMS have a smaller thymus than CTRL males. Change in spleen morphology has also been observed during stress response, especially after severe chronic stress, such as chronic social defeat stress in mice, where it can cause splenomegaly (Zhang et al., 2021; Wei et al., 2022). Inversely, we observed that NMS was associated with a reduction of spleen in males, which was associated with a lower proportion of T cells. This suggests that early life stress has distinct immune consequences when compared to chronic stress during yound adulthood, for instance, and that it could reduce their capacity to respond to viral infections. Thus, these results suggest that NMS influences lymphoid organs and that this effect is different depending on biological sex.

Interestingly, NMS males showed an expansion of macrophages population in lung at baseline. This is very interesting, given that AMs constitute the majority of immune cells present in BALs under homeostatic conditions (Qiu et al., 2012; Harris et al., 2019; Zeng et al., 2019). AMs are the first line of defense in the airways, while resident or interstitial macrophages are guardians of the lung interstitium and vasculature (Aegerter et al., 2022; Hou et al., 2021). They are tissue residents and have the role of maintaining homeostasis and acting as sentinels. Macrophages produce chemokines and cytokines that enable the recruitment and activation of neutrophils and monocytes. These cells play an important role in suppressing lung inflammation (Zhou et al., 2020). It is known that biological sex differences exist at baseline. For instance, the number of leukocytes, more precisely T and B lymphocytes and macrophages in peritoneal and pleural cavities of the lung is higher in female than male rats (Scotland et al., 2011). Furthermore, female macrophages exhibit enhanced phagocytosis and NADPH oxidase-mediated activity, suggesting that there is a sex difference in lung at baseline, leading to bacterial killing (Scotland et al., 2011). This can also lead to a more efficient innate response to pathogens in females after exposure to bacterial or viral components. Some authors have also demonstrated that LPS causes an infiltration of neutrophils, whereas Poly I:C led to a limited lymphocyte migration in mice (Starkhammar et al., 2012). Thus, this could suggest that AM of NMS males are dysfunctional, given their increased inflammatory response.

## 5 Conclusion

While this study vastly improved our understanding of the impact of early life stress on immune response, the exact underlying mechanisms linking early life stress and modulation of inflammatory response are still unclear, and are the aims of ongoing studies. Loss of contact with the mother is a risk factors for multiple diseases, such as cardiovascular and anxiety disorders, and causes distress in the newborn, exemplified by physiological alterations, such as heart rate and hormone levels (Vetulani, 2013; Shonkoff et al., 2009). Several studies have demonstrated that the maternal separation model increases the response of the HPA axis to stress in both young and adult animals (Tannenbaum et al., 1997; Miguel et al., 2019). In addition, several authors have demonstrated that the HPA axis plays a role in regulating the inflammatory response to infectious challenges (Yang et al., 2000; Bailey et al., 2003; Kioukia-Fougia et al., 2002). Thus, this article highlights how neonatal maternal separation as an early life stressor can impact innate and adaptive immune cells. Our data suggest that those exposed to NMS exert a sexually dimorphic effect and are at greater risk of respiratory tract infections. Further studies are needed to investigate microbial clearance and immune cell function, including AM and NK cells.

## CRediT authorship contribution statement

**Karine Bouchard:** Writing – review & editing, Writing – original draft, Project administration, Methodology, Investigation, Formal analysis, Data curation. **Dany Patoine:** Writing – review & editing, Supervision, Project administration, Methodology, Investigation, Formal analysis, Data curation, Conceptualization. **Joanny Roy:** Writing – review & editing, Supervision, Project administration, Methodology, Data curation. **Stéphanie Fournier:** Project administration, Methodology, Data curation. **David Marsolais:** Writing – review & editing, Funding acquisition, Conceptualization. **Richard Kinkead:** Writing – review & editing, Supervision, Methodology, Funding acquisition, Conceptualization. **Jean-François Lauzon-Joset:** Writing – review & editing, Writing – original draft, Supervision, Project administration, Funding acquisition, Data curation, Conceptualization.

## Declaration of competing interest

The authors declare the following financial interests/personal relationships which may be considered as potential competing interests:Jean-Francois Lauzon-Joset reports financial support was provided by Canadian Institutes of Health Research. Jean-Francois Lauzon-Joset reports financial support was provided by Québec Health Research Fund. Karine Bouchard reports financial support was provided by Québec Health Research Fund. If there are other authors, they declare that they have no known competing financial interests or personal relationships that could have appeared to influence the work reported in this paper.

## Figures and Tables

**figs1:**
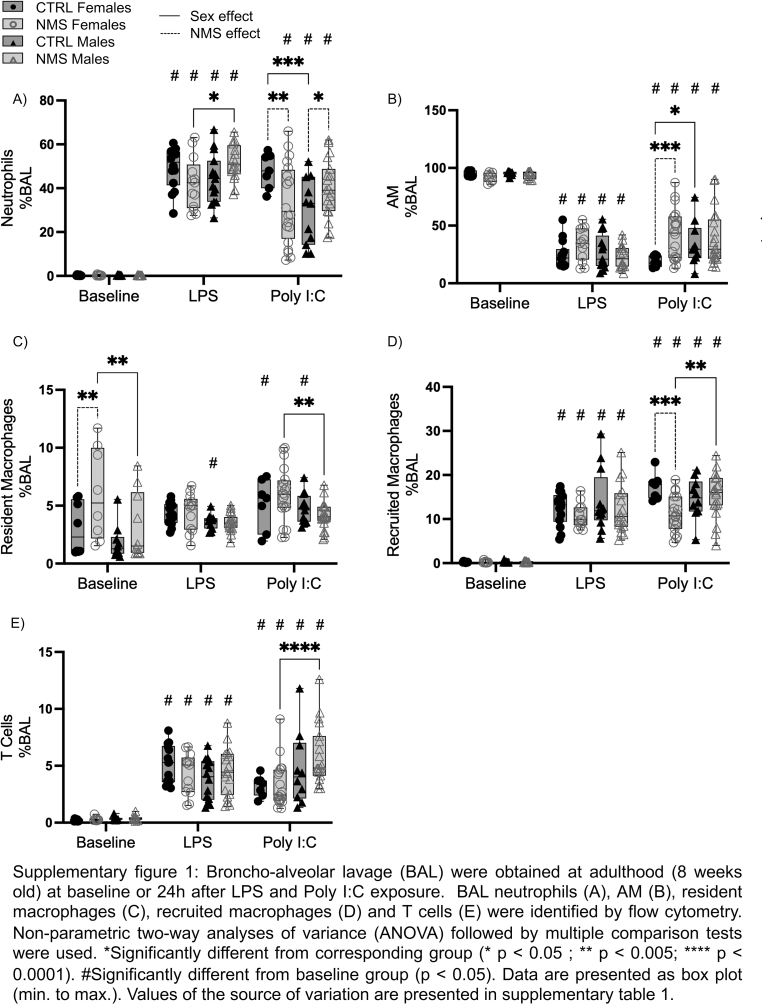


## Data Availability

Data will be made available on request.
