# Supplementary material for: Sex-specific impact of early life stress on adult lung inflammatory response after LPS and Poly I:C exposures
Source: Brain Behav Immun Health. 2026 Feb 23;53:101204. doi: 10.1016/j.bbih.2026.101204 (PMC12962109; doi:10.1016/j.bbih.2026.101204)
Supplement: Multimedia component 1 [file mmc1.pdf]

Supplementary Table 1

Source of variation (p-value) for figures 1-7 and supplementary figure 1.

		Interaction	Sex	NMS
Figure 1	B	0.9715	<0.0001	0.0788
	C	0.8263	0.0082	0.1027
	D	0.8986	0.0086	0.0345
	E	0.2126	0.5596	0.0739
	F	0.6108	0.0403	0.0083

		Interaction	Baseline/LPS/Poly I:C	Sex-NMS
Figure 2		0.0001	<0.0001	0.003
Figure 3	B	0.5027	<0.0001	0.3975
	C	0.1317	<0.0001	0.0633
	D	0.1191	<0.0001	0.6875
	E	0.6481	<0.0001	0.1978
	F	0.7952	<0.0001	0.212
Figure 4	A	0.2415	<0.0001	0.2872
	B	0.0025	<0.0001	0.1292
	C	0.0081	<0.0001	<0.0001
Figure 5	A	0.0014	<0.0001	0.0031
	B	0.1019	<0.0001	0.1554
	C	0.2876	<0.0001	0.9131
Figure 6	A	0.1116	<0.0001	0.1155
	B	<0.0001	<0.0001	<0.0001
Figure 7	A	0.4279	0.2046	0.7107
	B	0.089	<0.0001	0.0492
	C	0.0006	0.3138	0.3378
Supplementary Fig. 1	A	0.2119	<0.0001	0.0117
	B	0.1397	<0.0001	0.114
	C	0.0684	0.0009	<0.0001
	D	0.2295	<0.0001	0.0487
	E	0.0149	<0.0001	0.1385

Non-parametrical two-way analyses of variance (ANOVA) followed by multiple comparison test (Fisher's LSD) were used. Significantly different from corresponding group (*$p < 0.05$)
